# Clinical characteristics and outcomes of SARS-CoV-2-associated stroke: a large multicenter national cohort study

**DOI:** 10.31744/einstein_journal/2026AO2027

**Published:** 2026-06-23

**Authors:** João Brainer Clares de Andrade, Vinícius Viana Abreu Montanaro, Mayara Silva Marques, Kristel Larisa Back Merida, Rafaela Almeida Alquéres, Felipe Aydar Sandoval, Millene Rodrigues Camilo, Ahmad Ali El Madjdoub, Letícia Januzi de Almeida Rocha, Vinícius Luiz Cristofolini, Jackeline Viana da Silva, Rafael Paes Alves, Lorena Souza Viana, Felipe Ibiapina dos Reis, Patrícia Beatriz Christino Marinho, Pérola de Oliveira, Pedro Silva Correa de Magalhães, Vivian Dias Baptista Gagliardi, Sheila Cristina Ouriques Martins, Thais Leite Secchi, Octavio Marques Pontes-Neto, Iago Navas Perissinotti, Alan Alves de Lima Cidrão, Deborah Moreira Rangel, Rodrigo Bazan, Gabriel Pinheiro Modolo, Luana Aparecida Miranda Bonome, Felipe Araujo Rocha, Fabrício Oliveira Lima, Adriana Bastos Conforto, Daniela Laranja Gomes Rodrigues, Gisele Sampaio Silva

**Affiliations:** 1 Universidade Federal de São Paulo São Paulo SP Brazil Universidade Federal de São Paulo, São Paulo, SP, Brazil.; 2 Rede Sarah de Hospitais de Reabilitação Brasília DF Brazil Rede Sarah de Hospitais de Reabilitação, Brasília, DF, Brazil.; 3 Hospital Santa Cruz Rede D'or Curitiba PR Brazil Hospital Santa Cruz Rede D'or, Curitiba, PR, Brazil.; 4 Centro Universitário FMABC Neurology Service at the Central Emergency Room of São Bernardo do Campo Santo André SP Brazil Neurology Service at the Central Emergency Room of São Bernardo do Campo, Centro Universitário FMABC, Santo André, SP, Brazil.; 5 Universidade de São Paulo Ribeirão Preto SP Brazil Universidade de São Paulo, Ribeirão Preto, SP, Brazil.; 6 Hospital Santa Marcelina São Paulo SP Brazil Hospital Santa Marcelina, São Paulo, SP, Brazil.; 7 Universidade Federal de Alagoas Faculdade de Medicina Hospital Universitário Professor Alberto Antunes Maceió AL Brazil Hospital Universitário Professor Alberto Antunes, Faculdade de Medicina, Universidade Federal de Alagoas, Maceió, AL, Brazil.; 8 Centro Universitário São Camilo São Paulo SP Brazil Centro Universitário São Camilo, São Paulo, SP, Brazil.; 9 Hospital Israelita Albert Einstein São Paulo SP Brazil Hospital Israelita Albert Einstein, São Paulo, SP, Brazil.; 10 Universidade da Região de Joinville Joinville SC Brazil Universidade da Região de Joinville, Joinville, SC, Brazil.; 11 Hospital Municipal São José Joinville SC Brazil Hospital Municipal São José, Joinville, SC, Brazil.; 12 Santa Casa de São Paulo São Paulo SP Brazil Santa Casa de São Paulo, São Paulo, SP, Brazil.; 13 Hospital de Clínicas de Porto Alegre Neurology Department Porto Alegre RS Brazil Neurology Department, Hospital de Clínicas de Porto Alegre, Porto Alegre, RS, Brazil.; 14 Universidade de São Paulo Faculdade de Medicina Hospital das Clínicas São Paulo SP Brazil Hospital das Clínicas, Faculdade de Medicina, Universidade de São Paulo, São Paulo, SP, Brazil.; 15 Hospital Regional do Sertão Central Quixeramobim CE Brazil Hospital Regional do Sertão Central, Quixeramobim, CE, Brazil.; 16 Universidade Estadual Paulista "Júlio Mesquita Filho" Faculdade de Medicina de Botucatu Hospital das Clínicas Botucatu SP Brazil Hospital das Clínicas, Faculdade de Medicina de Botucatu, Universidade Estadual Paulista "Júlio Mesquita Filho", Botucatu, SP, Brazil.; 17 Hospital Geral de Fortaleza Fortaleza CE Brazil Hospital Geral de Fortaleza, Fortaleza, CE, Brazil.; 18 Hospital Alemão Oswaldo Cruz São Paulo SP Brazil Hospital Alemão Oswaldo Cruz, São Paulo, SP, Brazil.

**Keywords:** Severe acute respiratory syndrome-related coronavirus, Neurological manifestations, Risk factors, Stroke, SARS-CoV-2, Developing countries

## Abstract

**Objective:**

This study aimed to characterize the clinical, laboratory, and neuroimaging profiles of hospitalized Brazilian patients with confirmed SARS-CoV-2 and acute cerebrovascular events.

**Methods:**

This retrospective multicenter study included 374 patients from 17 stroke centers between March and November 2020. Eligible events were ischemic stroke, intracerebral hemorrhage, subarachnoid hemorrhage, and cerebral venous thrombosis. The stroke subtypes were further classified using the TOAST, Oxfordshire, and SMASH-U criteria. Demographics, National Institutes of Health Stroke Scale (NIHSS) scores, laboratory results, imaging findings, complications, and outcomes were analyzed. Multivariate logistic regression was used to assess the predictors of in-hospital mortality and good functional outcomes (modified Rankin Scale; mRS 0- 2).

**Results:**

Ischemic stroke accounted for approximately 83% of all stroke cases. The median patient age and NIHSS score at admission were 66 years and 10, respectively. The major complications included pneumonia (61%), renal failure (35%), and sepsis (30%). The in-hospital mortality rate reached 31%, and only 28% of the patients achieved good functional outcomes. Higher NIHSS scores and D-dimer levels were independently associated with worse outcomes. Notably, the median time from stroke onset to admission (4.4 h) was shorter than the pre-pandemic average.

**Conclusion:**

In this national cohort, SARS-CoV-2-associated stroke was severe, predominantly ischemic, and commonly complicated by systemic organ dysfunction. D-dimer levels emerged as a significant prognostic biomarker of mortality and poor functional outcomes. Contrary to earlier assumptions, stroke admission delays were not prolonged during the pandemic. These findings highlight the need for integrated stroke care in patients with COVID-19 and inform future management strategies.

## In Brief

In a multicenter Brazilian cohort of 374 hospitalized patients with COVID-19 and acute cerebrovascular events, ischemic stroke predominated (83.7%) with severe outcomes and 31% in-hospital mortality. Only 28% of patients achieved good functional status. D-dimer level emerged as an independent predictor of mortality (OR=1.036, p=0.04), while NIH Stroke Scale score predicted poor functional outcomes (OR=1.15, p<0.001). Contrary to early pandemic concerns, stroke admission delays were not prolonged compared to pre-pandemic averages, with a median onset-toadmission time of approximately 4 h.

## Highlights

■ SARS-CoV-2 patients had 83.7% ischemic stroke and 39% in-hospital mortality rates.
■ D-dimer levels independently predict mortality in patients with COVID-19 and stroke.
■ Patients with COVID-19 and stroke have high complication rates: pneumonia 61%, renal failure 35%, sepsis 30.3% sepsis.
■ Time from symptom onset to admission was shorter than the pre-pandemic average.

## Graphical abstract

**Figure:**
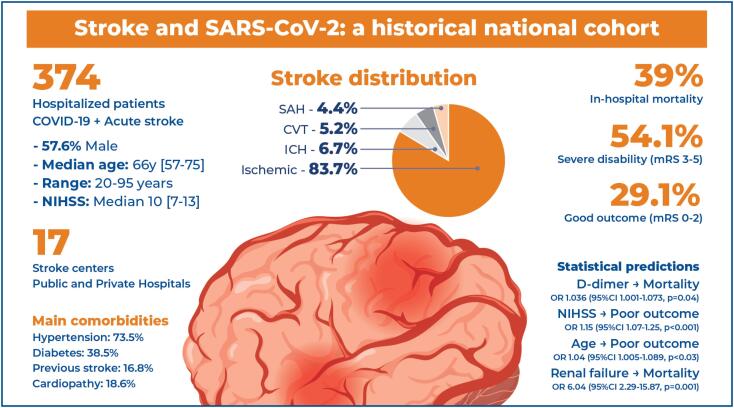

## INTRODUCTION

Several studies have demonstrated that severe acute respiratory syndrome coronavirus 2 (SARS-CoV-2) infection can induce neurological manifestations, ranging from mild to severe, potentially involving the central nervous system (CNS), peripheral nervous system (PNS), and skeletal muscles^(1,2)^. Notably, stroke has been described as a complication of severe COVID-19 and an initial indicator of the disease. These cerebrovascular events may be caused by endothelial injury, hypercoagulable state, or cardiogenic embolism^(2,3)^. Although the exact mechanism through which SARS-CoV-2 affects the nervous system remains unclear, several potential explanations have been proposed. Consequently, various clinical, laboratory, and imaging biomarkers have been explored to track and understand neurological complications^(4)^. Additionally, outside the context of a pandemic, outbreaks of coronavirus infections have been documented as understanding their neurological impact can inform new therapeutic and prognostic strategies.

Historical data from the pre-vaccine era provide valuable insights into the behavior of SARS-CoV-2 infections, aiding in understanding viral variant dynamics and the pathophysiological mechanisms underlying long-term outcomes ("Long COVID")^(5,6)^. However, national reports on COVID-19 neurological manifestations are still scarce, especially in countries with high case counts. In Brazil alone, nearly 40 million COVID-19 cases were reported (fatality rate of approximately 2%), making it an important setting to study the neurovascular impacts of COVID-19 in a diverse population.^(7)^ Notably, the COVID-19 pandemic has significantly affected stroke care. Studies in Brazil and other countries have reported substantial reductions in stroke hospitalizations (e.g., a 32% decline in ischemic stroke admissions in São Paulo) during the first wave of the pandemic, along with the need to reorganize emergency services to accommodate patients with COVID-19^(8-10)^. These systemic factors underscore the need to interpret the profiles and outcomes of COVID-19-associated stroke.

## OBJECTIVE

We aimed to retrospectively describe the clinical, laboratory, and neuroimaging features of in-hospital patients with SARS-CoV-2 and acute cerebrovascular disease, compare these features across stroke subtypes, and analyze outcome predictors to better characterize the intersection of COVID-19 and stroke in a middle-income country during the pre-vaccination period of the pandemic.

## METHODS

### Study design and population

This multicenter, observational, retrospective study used data from a population-based stroke registry that includes 17 comprehensive stroke centers across Brazil. The study was conducted from March to November 2020, corresponding to the later phase of the first COVID-19 wave in Brazil and the pre-vaccination era.

### Inclusion criteria

We included consecutive hospitalized patients aged ≥18 years who tested positive for SARS-CoV-2 (via RT-PCR or serology) within 14 days of stroke onset and had a confirmed acute cerebrovascular event. Cerebrovascular diagnoses included ischemic stroke, intracerebral hemorrhagic stroke, subarachnoid hemorrhage (SAH), or cerebral venous thrombosis (CVT). Patients were evaluated by a neurologist within 24 h of admission and underwent at least one neuroimaging study within 7 days of the onset of neurological symptoms. We excluded patients with inconsistent diagnoses, refusal to undergo neurological evaluation, and missing key initial data.

### Data Collection

De-identified data were collected by trained local investigators (stroke neurologists, residents, or research staff) at each center using a standardized electronic case report form. The collected variables included sociodemographic information, pre-existing comorbidities (e.g., hypertension, diabetes, atrial fibrillation, obesity, and smoking status), COVID-19-related information (symptoms, home isolation status, and family COVID exposure), and stroke-specific data. The stroke onset time, mode of hospital arrival, and time from stroke onset to hospital admission were also recorded. We assessed initial stroke severity using the NIH Stroke Scale (NIHSS) at admission and pre-stroke disability using the modified-Rankin Scale (mRS). All patients underwent at least one brain imaging assessment (CT and/or MRI). Stroke subtypes were classified as ischemic stroke, intracerebral hemorrhage, SAH, or CVT. Ischemic stroke was further categorized using the Oxfordshire Community Stroke Project (OCSP) classification into total anterior circulation infarcts, partial anterior circulation infarcts, lacunar infarcts, and posterior circulation infarcts. Ischemic stroke etiology was determined using the Trial of ORG 10172 in Acute Stroke Treatment (TOAST) criteria when data were available. The causes of intracerebral hemorrhage were categorized using the SMASH-U classification, and SAH etiologies (aneurysmal *versus* non-aneurysmal) were noted. We also documented in-hospital medical complications (pneumonia, sepsis, pulmonary embolism, and acute renal failure), treatments for COVID-19 (e.g., use of steroids, anticoagulants, and antivirals), and acute stroke therapies (IV thrombolysis or mechanical thrombectomy, when applicable). Functional outcomes at hospital discharge were measured using the mRS, and vital status (in-hospital survival or death) was recorded.

### Statistical analysis

Continuous variables are reported as mean±standard deviation (SD) or median [interquartile range, IQR], as appropriate, and categorical variables as frequencies (%). Group comparisons (ischemic *versus* hemorrhagic strokes) were performed using Student's t-test or Mann- Whitney U test for continuous data (depending on normality), and χ^2^ or Fisher's exact test for categorical data. Statistical significance was set at p<0.05. Multivariate analysis was conducted using logistic regression to identify the independent predictors of outcomes. Two logistic regression models were constructed to assess the predictors of good functional outcomes at discharge (mRS score 0- 2) and in-hospital mortality. A stepwise backward-elimination approach was used to refine the model. We assessed the goodness-of-fit of the model using the Hosmer- Lemeshow test and ensured no major collinearity between covariates. Adjusted odds ratios (OR) with 95% confidence intervals (95%CI) were calculated for significant predictors. In the multivariate logistic regression models, we adjusted for classical stroke risk factors including age, hypertension, diabetes mellitus, atrial fibrillation, and previous stroke, in addition to acute severity markers (NIHSS) and inflammatory/coagulation biomarkers (D-dimer). Given that all patients in this cohort had confirmed SARS-CoV-2 infection by design, we could not assess whether COVID-19 itself was an independent risk factor for stroke occurrence or poor outcomes. The variables entered into the multivariate models included age, baseline hypertension, diabetes mellitus, atrial fibrillation, previous stroke, NIHSS score at admission, and D-dimer level. A comprehensive univariate analysis of all potential predictors is presented in Table S1, Supplementary Material. Variables with p<0.10 in univariate analysis were considered for entry into stepwise multivariate models. The final models demonstrated adequate statistical power with events per variable (EPV) ratios of 16.7 for mortality (117 deaths/7 variables) and 13.6 for favorable outcomes (95 events/7 variables), both exceeding the recommended minimum of 10 EPV. The variant inflation factors for all covariates were <2.5, indicating no significant multicollinearity.

All analyses were performed using SPSS version 28.0 (IBM Corp., Chicago, IL, USA).

### Ethical approval

This study was approved by the Institutional Review Board of the local hospital (*Universidade Federal de São Paulo*, CAAE: 31351220.8.1001.5505; 4.354.729). All the national ethical requirements were met to support this study. All data used in the analysis are presented in tables and. The requirement for individual informed consent was waived owing to the retrospective, observational design with de-identified data.

## RESULTS

This study included 374 patients with cerebrovascular disease who tested positive for SARS-CoV-2 between March and November 2020. The patient ages ranged from 20- 95 years (median 66 [57-75]), with a predominance of men (57.6%). The mean time from SARS-COV-2 symptoms to neurological impairment was 6±8.9 days, and 62.2% patients exhibited SARS-COV-2 symptoms at hospital admission. Stroke symptom recognition by family members occurred in 28.8% cases. The treatment strategies used for SARS-COV-2 infection were macrolides (34.5%), steroids (21.7%), heparin (9.2%), and chloroquine (6.9%). The most common clinical complication was pneumonia (61%), followed by acute renal failure (35%), sepsis (30.3%), cardiac arrest (27.9%), venous thromboembolism (9.1%) and delirium (8.2%). Among the patients, 288 (83.7%), 23 (6.7%), 18 (5.2%), and 15 (4.4%) patients had a final diagnosis of ischemic stroke, intracerebral hemorrhage, cerebral venous thrombosis, and subarachnoid hemorrhage, respectively. Demographic and clinical baseline characteristics are shown in table 1.

**Table 1:** Demographics and clinical characteristics

Variable		All patients (n=374) (±SD) [IQR]	Ischemic Stroke (n=312) (±SD) [IQR]	Intracerebral hematoma (n=29) (±SD) [IQR]	Subarachnoid hemorrhage (n=15) (± SD) [IQR]	Cerebral venous thrombosis (n=18) (±SD) [IQR]
Age, years		65 [55-75]	67 [57-76]*	63 [51-67]	63 [56-71]	48 [26-62]*
Male, %		56.9	60.9*	48.2	13*	38.9
Admitted to a private hospital, %		9	8.3	13.6	7.1	16.7
ASPECTS on admission, points		-	10 [10, 7]	-	-	-
NIH Stroke Scale on admission, points		10 [4, 19]	10 [5, 19]*	9 [4, 14]*	-	-
Wake-up symptoms, %		11	12.8*	3.4	0	0
Without time for reperfusion therapy, %		-	42.9	-	-	-
Time from stroke onset to admission, minutes		655 (2594)	954.7 (230)*	480 (300)		
Presence of COVID-19 symptoms at neurological symptoms onset, %		62.2	64.2	47.8	40	66.7
Time from COVID-19 symptoms to neurological symptoms, days		6 (8.9)	6 (9)	4.6 (7.6)	6.9 (10.5)	8.2 (8.4)
Fluctuating symptoms, %		7.5	6.1*	3.4	6.7	38.9*
Home isolation, %		36.6	37.9	34.8	26.7	27.7
Identification of CVD symptoms by familiars, %		30.2	30.4	44.2	20	11.1
OCSP classification on admission						
	LACI	-	22.6	-	-	-
	PACI	-	26.8	-	-	-
	TACI	-	34.3	-	-	-
	POCI	-	14.0			
SMASH-U Classification, %						
	Systemic diseases	-	-	8	-	-
	Medication	-	-	20	-	-
	Amyloid angiopathy	-	-	0	-	-
	Structural	-	-	8	-	-
	Hypertensive	-	-	24	-	-
	Undetermined	-	-	40	-	-
Artery involvement, %						
	MCA	-	72.2	-	-	-
	ACA	-	2	-	-	-
	PCA	-	2.4	-	-	-
	Basilar	-	9.2	-	-	-
	Anterior and posterior	-	6.2	-	-	-
	Two or more arteries	-	13.4	-	-	-
	TOAST SSS - Etiology, %	-		-	-	-
	Cardio-aortic	-	19.8	-	-	-
	Large vessels	-	19.8	-	-	-
	Small vessels	-	7.5	-	-	-
	Others	-	6.4	-	-	-
	Undetermined	-	21.4	-	-	-
	Incomplete evaluation		25.1			
Comorbidities, %						
	Arterial hypertension	73.5	75	78.9	64.3	50
	Diabetes	38.5	41.5*	26.3	21.4	21.4
	Atrial fibrillation	4.1	5.3*	4.3	0	0
	Previous Stroke	16.8	19.7*	0	7.1	0
	Obesity	10.7	9.8	10.5	21.4	14.3
	Current smoking	11	11.1	10.5	21.4	0
	Cardiopathy	18.6	19.7	26.3	7.1	0
Laboratory findings on admission						
	Hemoglobin, g/L	12 (8.6)	12.1 (9.1)	11.7 (3.6)	10.8 (4.1)	11.5 (5.5)
	WBC, /mm^3^	9710 (6343)	9378 (6281)*	11300 (6695)	13898 (6072)*	9497 (6053)
	Platelets, /mm^3^	268149 (159898)	287997 (9438)*	195647 (110312)*	242866 (135852)	240288 (151064)
	Creatinine, mg/dL	1.6 (8.5)	1.6 (9.1)	2.3 (4.1)*	1.9 (3.6)	1.2 (1.4)
	Urea, mg/dL	44.6 (40.4)	43.4 (38.5)	58.4 (51.2)	57.6 (58.6)	37 (34)
	D-Dimer, ng/ml	3.9 (17.6)	4 (19)**	2.7 (5.3)	4 (7.6)	2.5 (4.8)
	LDH, mg/dL	183 (454)	169 (295)	410 (1401)	205 (256)	96 (155)
	Thrombin Time/INR, s	0.98 (0.85)	0.96 (0.83)	1.16(1.4)	0.97 (0.5)	1.04 (0.4)*
	aPTT/INR, s	0.64 (0.56)	0.6 (0.53)	0.75 (0.9)	0.82 (0.47)	0.83 (0.48)
	Sedimentation rate, seconds	4.4 (18)	3.6 (15)	4 (19)	5.6 (22)	15.6 (38.7)*
Neurological and clinical complications, %						
	Hemorrhagic transformation	-	11.1	-	-	-
	Recurrent ischemia	-	2.8	-	-	-
	Delirium	8.2	8.7	6.2	0	20
	Pneumonia	61	63.1	69	36.4	40
	Acute renal failure	35	33.3	50	36.4	40
	VTE or DVT	9.1	7.6	18.7	9.1	20
	Sepsis	30.3	31	18.7	36.4	30
	Cardiac arrest	27.9	26.9	31.2	27.3	40
In-hospital treatment, %						
	Chloroquine	6.9	7.1	6.2	8.3	0
	Steroids	21.7	21.4	18.8	41.7	9.1
	Heparin	9.2	7.2*	25*	0	36.4*
	Macrolides	34.5	34.3	31.2	41.7	36.3
	Combined treatment	30	30	31.2	33.3	27
Clinical outcomes, %						
	mRS 0-2	29.1	27.2	30	21.4	55.5*
	mRS 3-5	55	59*	47	25*	10*
	In-hospital death	31	32.8*	44.5	71.4*	38.8

* p≤0.05 compared to other groups

** p≤0.05 compared to patients with ICH.

MCA: middle cerebral artery; PCA: posterior cerebral artery; ACA: anterior cerebral artery; OCSP: Oxfordshire Community Stroke Project; LACI: lacunar infarct; PACI: partial anterior circulation infarct; TACI: total anterior circulation infarct; POCI: posterior anterior circulation infarct; TOAST SSS: Trial of Org 10172 in Acute Stroke Treatment and Stop Stroke Study; WBC: white blood cell; LDH: lactate dehydrogenase; aPTT: active partial thromboplastin time; CVD: cerebrovascular disease; VTE: venous thromboembolism; ICH: Intracerebral hematoma; DVT: deep vein thrombosis; mRS: Modified Rankin Scale.

Baseline cardiovascular medication use (antiplatelets, anticoagulants, and statins) prior to stroke was not systematically collected in this registry and was therefore not reported.

Patients with ischemic stroke were older and had a higher incidence of diabetes (41.5%), atrial fibrillation (5.3%), and a previous stroke (19.7%) (Table 1). The median NIHSS on admission was 10 [7, 13], and the mean time from stroke onset to admission was 954.7 (±230) min, with 13.9% of wake-up strokes. D-dimer (p=0.02) and platelet counts (p=0.04) were higher in patients with ischemic stroke than in those with other cerebrovascular diseases.

All 17 participating stroke centers were capable of performing thrombolysis throughout the study period. Among 312 patients with ischemic stroke, 161 (51.6%) did not receive reperfusion therapy. Of these, 80 (49.7%) arrived outside the therapeutic time window, whereas 81 patients (50.3%) presented within the appropriate time window, but did not receive reperfusion. Analysis of the 81 patients revealed several contributing factors: 28.4% had mild strokes (NIHSS ≤4), for which the risk of reperfusion therapy may outweigh potential benefit according to current guidelines. Next, 77 patients (24.7%) received reperfusion therapy; 64 received intravenous thrombolysis with recombinant tissue plasminogen activator (rtPA), five underwent mechanical thrombectomy alone, seven received combined intravenous thrombolysis plus thrombectomy (bridging therapy), and one patient received Tenecteplase.

The median NIHSS score at admission was 10 points [7- 13], indicating moderate overall stroke severity. Patients with ischemic and hemorrhagic strokes had similar NIHSS median scores (both around 9- 10), whereas those with CVT tended to have lower NIHSS scores (many patients with CVT had no focal NIHSS deficits; data not shown). The median time from stroke symptom onset to hospital arrival (pre-hospital phase) was 4.3 h (IQR, 2- 12 h) for the entire cohort, although this varied by stroke type: ischemic and hemorrhagic stroke and cerebral venous thrombosis had a median onset-to-door time of 4.5 h, 2.7 h, and 48 h, respectively. Wake-up stroke (symptom onset during sleep with an unknown exact time) accounted for 11.0% of the cases. Detailed in-hospital time metrics (such as door-to-needle or door-to-groin times) were not systematically collected across all the centers in this observational registry. Notably, this median onset-to-door time was shorter than the pre-pandemic benchmarks reported in Brazil, suggesting that despite the pandemic, many stroke patients reached hospitals relatively quickly. However, approximately one-third of the patients with ischemic stroke (36.6%) arrived outside the thrombolysis/thrombectomy time window and did not receive reperfusion therapy. Among patients with ischemic stroke, 42.9% were ineligible for acute reperfusion because of delayed presentation. Anterior circulation infarcts (especially total anterior circulation infarcts, 34.3%) were common in the ischemic stroke subset (Table 1), and 13.2% of these patients had multifocal strokes involving at least two arterial territories (suggestive of embolic or coagulopathic mechanisms). Among patients with hemorrhagic strokes, 40% had an undetermined etiology according to the SMASH-U criteria (no clear hypertensive or structural cause), and 35.7% of SAH cases had no identified aneurysm (presumed non-aneurysmal SAH).

Laboratory parameters on admission revealed elevated levels of inflammatory and coagulopathic markers in several patients. The mean D-dimer level was markedly high (overall mean 3,054ng/mL, median 1,200 ng/mL; normal <500ng/mL), and D-dimer values were significantly higher in patients with ischemic stroke than in those with hemorrhagic stroke, SAH, or CVT (p=0.02). Additionally, the mean platelet count was high in the ischemic and nonischemic groups (p=0.04). No significant differences were observed in other laboratory values, such as leukocyte count or C-reactive protein level, between the stroke subtypes (not shown), although most patients had some inflammatory abnormalities. The COVID-19 treatment strategies varied: 34.5%, 21.7%, 9.2%, 9.2% patients received macrolide antibiotics, corticosteroids, therapeutic anticoagulation (heparin), and chloroquine or hydroxychloroquine during hospitalization (reflecting practices in early 2021), respectively.

**Figure 1:**
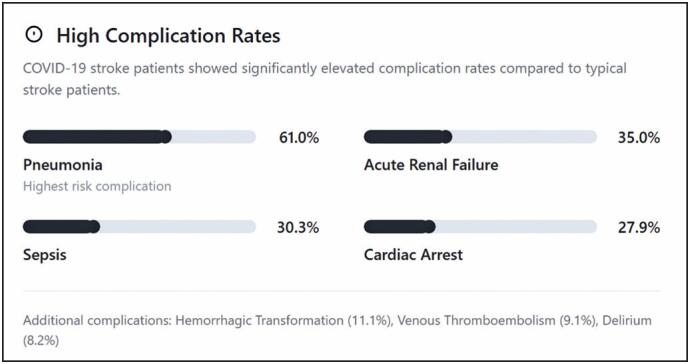
Clinical complications

The clinical outcomes of the study cohort were poor. Overall, 54.1% of the patients had severe functional impairment (mRS score 3- 5) at discharge, and the in-hospital mortality rate was 31%. This indicates that only approximately one-quarter of the patients achieved a favorable outcome (mRS 0- 2) at the time of discharge. Figure 1 illustrates the distribution of the functional outcomes. Major in-hospital complications were common; 61% of the patients developed pneumonia, 35% experienced acute kidney injury or renal failure, and 30.3% developed sepsis during their hospital stay. Other complications included cardiac arrest (27.9%), venous thromboembolism (deep vein thrombosis or pulmonary embolism), (9.1%), and delirium (8.2%). Patients often experienced multiple concurrent complications. Only 8% of the patients required decompressive craniectomy for malignant edema (all ischemic stroke cases) (Figure 1). At discharge, 15.9% of the survivors were independent (mRS 0- 2), 24.4% had a moderate disability (mRS 3- 4), 21.6% were severely disabled (mRS 5), and 38.1% had died (mRS 6).

**Figure 2:**
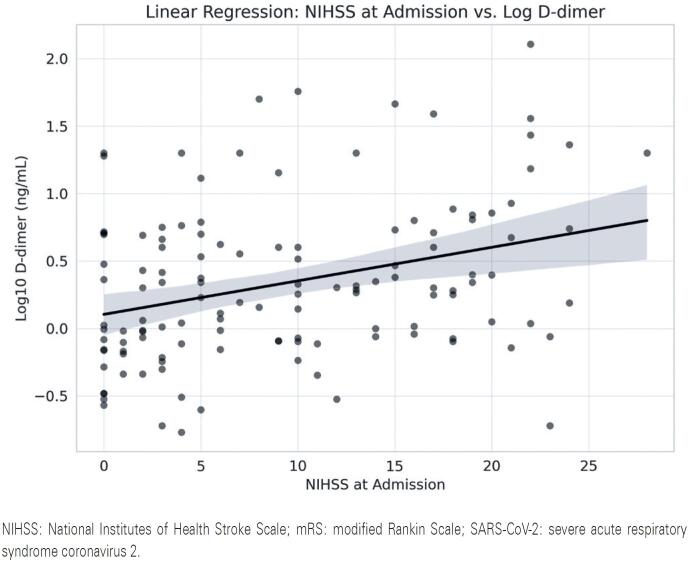
Linear regression between NIHSS at admission and log-transformed D dimer levels

NIHSS: National Institutes of Health Stroke Scale; mRS: modified Rankin Scale; SARS-CoV-2: severe acute respiratory syndrome coronavirus 2.

In the univariate analysis, factors associated with in-hospital mortality included older age, higher NIHSS score on admission, occurrence of renal failure, and elevated D-dimer levels. Conversely, prior use of anticoagulants or antiplatelet agents and ischemic versus hemorrhagic stroke type were not significantly associated with mortality. Subsequently, multivariate logistic regression analysis was performed by adjusting for confounders. The results revealed that a higher D-dimer level at admission was independently associated with increased odds of in-hospital death (OR=1.036 per 1-unit increase; 95%CI=1.001- 1.073; p=0.04; Figure 2). Admission NIHSS score and the development of acute renal failure were also independent predictors of mortality: each point increase in the NIHSS score corresponded to an OR of ∼1.12 for death (95%CI=1.06- 1.19, p=0.004), and patients who experienced renal failure had a markedly higher risk of dying (OR=6.04, 95%CI=2.29- 15.87, p=0.001). No other variables (age, stroke subtype, or other complications) retained independent significance for mortality after adjustment. Notably, the p-value for the D-dimer effect was only 0.04, reflecting a marginal significance level. Moreover, no correction for multiple comparisons was applied; therefore, this finding should be interpreted with caution. After adjusting for cardiovascular risk factors (age, hypertension, diabetes mellitus, atrial fibrillation, and previous stroke) in the multivariate models, independent predictors of mortality were NIHSS score at admission and D-dimer level.

In the univariate analysis, classical risk factors showed variable associations; hypertension showed a trend toward significance (p=0.06), while diabetes (p=1.00), atrial fibrillation (p=1.00), and previous stroke (p=0.39) did not reach statistical significance as individual predictors. Therefore, we performed a sensitivity analysis after excluding 53 patients (14.2%) with previous cerebrovascular events. In this subgroup analysis (n=268), the independent predictors of mortality remained unchanged: NIHSS at admission (OR per point 1.14, 95%CI=1.07-1.21, p<0.001) and D-dimer level (OR=per ng/ml 1.04, 95%CI=1.01-1.08, p=0.038) remained significant predictors of mortality even after adjustment for age, hypertension, diabetes, and atrial fibrillation. The magnitude and direction of the associations were consistent with the primary analysis, suggesting that the inclusion of patients with prior stroke did not materially bias our conclusions.

Scatter plot with linear regression line showing the relationship between stroke severity at admission, as measured by the National Institutes of Health Stroke Scale (NIHSS), and plasma D-dimer levels (log_10_-transformed) in patients with acute cerebrovascular events and SARS-CoV-2 infection. A weak positive linear association was observed, suggesting that patients with higher NIHSS scores had elevated D-dimer levels upon admission. D-dimer is a marker of coagulation activation and systemic inflammation and is commonly elevated in severe COVID-19.

**Figure 3:**
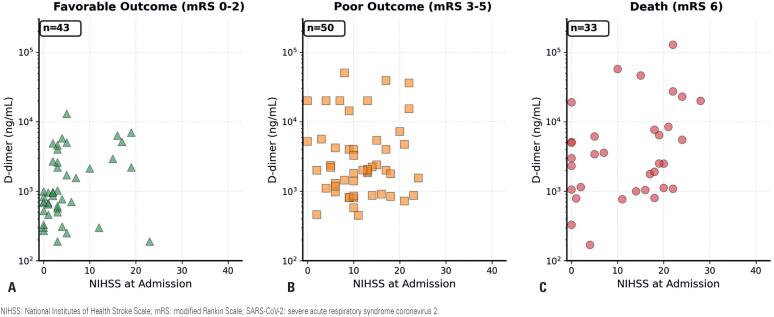
Relationship between NIHSS and D-dimer by functional outcome

NIHSS: National Institutes of Health Stroke Scale; mRS: modified Rankin Scale; SARS-CoV-2: severe acute respiratory syndrome coronavirus 2.

Multivariate analysis for good functional outcomes (mRS 0- 2 at discharge) revealed that admission NIHSS score and D-dimer levels were independent predictors. A higher NIHSS score was strongly predictive of worse functional outcomes (OR=0.88 per point for achieving mRS 0- 2, p=0.001, indicating that an increase in the NIHSS score reduced the odds of a good outcome by 12% (Figure 3). Similarly, higher D-dimer levels were associated with lower odds of a good outcome (OR=0.86 per unit, 95%CI=0.76- 0.99, p=0.03), which is consistent with the fact that greater coagulopathy/inflammation portends worse recovery. Age was also significantly associated with poor outcome in univariate analysis; however, after adjusting for NIHSS and D-dimer, age had a more modest effect (OR=0.96 per year for good outcome, p=0.08) and was not statistically significant in the final model. Additionally, the use of any COVID-19 directed therapies (such as steroids or anticoagulants) was not significantly related to the outcomes or mortality (p>0.1 for all adjusted models).

**Figure 4:**
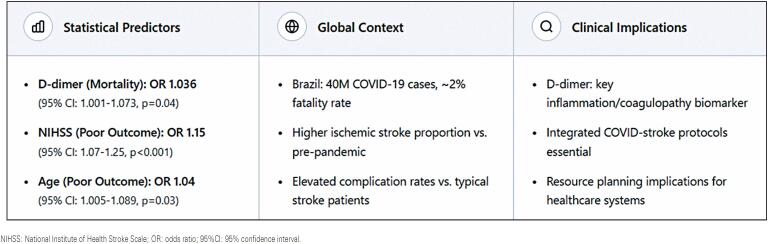
Predictive factors and clinical implications

NIHSS: National Institute of Health Stroke Scale; OR: odds ratio; 95%CI: 95% confidence interval.

The summary of predictors, context and clinical implications is portrayed in figure 4.

Relationship between the NIHSS score and D-dimer levels stratified by functional outcome at discharge. Three-panel scatter plots showing the association between stroke severity at admission (National Institutes of NIHSS, x-axis) and D-dimer levels (ng/ml, y-axis, log scale), stratified by functional outcomes. Panel A: Favorable outcome (mRS 0-2, n=95). Panel B: Poor outcomes (mRS 3-5, n=115). Panel C: Death (mRS 6, n=117). The progressive increase in both NIHSS and D-dimer values is evident across the worsening outcome categories, with patients who died showing the highest values for both biomarkers.

## DISCUSSION

In this large multicenter cohort study, we observed that COVID-19 in hospitalized patients was frequently associated with acute ischemic stroke (>83% of cases), with relatively fewer hemorrhagic strokes, SAH, or CVT. This distribution was skewed slightly more toward ischemic stroke compared to that in pre-pandemic stroke epidemiology in Brazil, where ischemic stroke typically accounted for approximately 80% of cases.^(10,11)^ The median age and sex distribution in our COVID- stroke cohort were similar to those in non-COVID stroke populations in middle-income countries, although a trend toward fewer patients with a history of prior stroke or known atrial fibrillation than in historical stroke cohorts was observed.^(7,11)^ This suggests that some COVID-related strokes occurred in individuals without traditional cerebrovascular risk factors, which is consistent with early reports of COVID-19 causing stroke in younger, healthier patients, possibly via novel mechanisms (such as coagulopathy and endothelialitis).^(12-14)^ Nonetheless, hypertension and diabetes remained highly prevalent in our cohort, reinforcing the idea that conventional risk factors play a role in stroke patients with COVID-19.

Notably, our analyses showed high rates of in-hospital complications and mortality. Our observed in-hospital mortality rate of 39% was substantially higher than the typical mortality rate of patients with stroke prior to COVID-19. Specific Brazilian stroke registries have reported in-hospital mortality rates of approximately 15- 20%, although these figures come from select centers and may not be generalizable to all healthcare settings or geographic regions across Brazil. Additionally, the observed mortality rate is higher than the 12- 16% in-hospital stroke mortality reported during the pandemic in at least one general stroke cohort in a Brazilian city (Joinville).^(10,11)^ This discrepancy underscores the fact that our study specifically examined the subset of patients with both stroke and COVID-19. Accordingly, this rate appears to be consistent with international series that have reported very high mortality in COVID-associated stroke. For example, a North American registry reported an approximately 39% in-hospital mortality in patients with COVID-19 ischemic stroke.^(15)^ Generally, evidence suggests that COVID-19 and stroke lead to substantially worse outcomes than does stroke in COVID-negative patients.^(16)^ The high mortality in our cohort likely reflects both the direct impact of viral infection and the severity of the stroke itself. Many patients experience multi-organ failure (as indicated by high rates of sepsis, renal failure, and respiratory complications), which undoubtedly contributes to the high fatality rate. Additionally, hospital resources may have been strained during the peak of the pandemic, which could have indirectly affected stroke care outcomes.

Our multivariate analysis identified D-dimer level as an independent predictor of mortality, even after controlling clinical factors. This finding is consistent with a growing body of evidence linking elevated D-dimer levels and coagulopathy markers to worse outcomes in COVID-19 and stroke populations.^(4,13,17)^ High D-dimer in patients with COVID-19 likely reflects a state of hypercoagulation and thrombus formation (from widespread intravascular clotting or severe inflammation). Stroke may indicate a more extensive infarction or an underlying propensity for thrombosis (e.g., venous thromboembolism or disseminated intravascular coagulation). Additionally, previous studies have found that D-dimer levels are associated with stroke severity and can predict mortality in patients with ischemic stroke and COVID-19.^(4,5)^ Thus, our results reinforce that D-dimer level is a useful prognostic biomarker in this specific patient population. Clinically, this might justify more aggressive monitoring or prophylactic anticoagulation strategies, although our data did not show a clear benefit from therapeutic heparin use (possibly owing to confounding by indication). Notably, the average time from stroke onset to hospital admission was shorter in our patients with COVID-19-associated stroke than the reported pre-pandemic averages.^(10)^ However, the statistical significance of D-dimer levels as a predictor of mortality was marginal (p=0.04), and no correction for multiple comparisons was applied in our exploratory analysis. Therefore, although this finding aligns with the established biological mechanisms of COVID-19-related coagulopathy, it should be validated in larger, independent cohorts before being incorporated into clinical decision-making algorithms.

We originally hypothesized that pandemic-related factors, such as patients avoiding hospitals because of fear of infection or healthcare system overload, would lead to delayed presentation and treatment of stroke.^(9,18- 21)^ aHowever, our findings suggest that the median onset-to-door time (∼4.4 h) was relatively brisk. One explanation for this finding is that some strokes in patients with COVID-19 occurred in the hospital or under medical supervision (e.g., patients already hospitalized for COVID-19 who then suffered a stroke, which would drastically shorten the delay time).^(21,22)^ Another explanation is that the increased vigilance during the COVID-19 surge, both by patients/families and emergency medical services, may have led to faster recognition and response when neurological symptoms appeared in the COVID-19 context. Additionally, the overall volume of emergency room for non-COVID conditions was lower than normal during the lockdown, potentially allowing patients with stroke who did come in to be triaged and treated more rapidly than usual.^(18,19)^ Moreover, several patients (62.2%) in this study had severe COVID-19 symptoms that prompted early hospital arrival and in whom stroke occurred shortly after admission. Nevertheless, our data suggest that the feared substantial delays in stroke care during COVID-19 might not have occurred universally, at least in the participating centers, and in fact, the median door times did not worsen. This contrasts with some early reports during the pandemic that showed decreased stroke admissions and procedure volumes due to patients avoiding hospitals.^(18,21,22)^

Our findings are in line with the experience in Joinville, Brazil, where local stroke network-maintained performance metrics during the pandemic and overall stroke care times were preserved.^(10)^ Another notable observation was that only a small fraction of all hospitalized patients with COVID-19 experienced stroke. While we did not have data on the total COVID-19 admissions for each hospital, external data suggest that the incidence of stroke among hospitalized patients with COVID-19 was on the order of a few percent.^(23)^ While a meta-analysis across continents reported an incidence rate of approximately 3.3%, with South America at the highest end (8.9%),^(24)^ our multi-center registry focused on those who had a stroke, providing insights into this severe subset.

Although stroke in patients with COVID-19 is relatively infrequent, we observed severe outcomes, which highlights a major challenge that these are rare but extremely high-risk patients. Consequently, identifying patients with COVID-19 at risk of stroke (e.g., those with high D-dimer levels, extreme inflammation, or certain comorbidities) could allow for preventive measures in the future.^(4,13,25)^ From a pathophysiological standpoint, our findings support the proposed mechanisms underlying COVID-19-associated stroke. The predominance of large anterior circulation and multifocal infarcts in patients with ischemic stroke suggests an embolic and hypercoagulable etiology in many cases.^(23,24,26)^ This is consistent with the concept that COVID-19 induces a thromboinflammatory state with endothelial dysfunction, cytokine storm, platelet activation, and microthrombus formation.^(12)^ Notably, 13% of the patients with ischemic strokes in this study had multiple territories involved, and cryptogenic stroke (stroke of undetermined etiology) was common- findings echoed in other series where cryptogenic mechanisms (perhaps undetected coagulopathy or endotheliopathy) were prevalent. The occurrence of CVTs and non-aneurysmal SAH in our cohort, although minor, hints at the unique COVID-19 effects on the coagulation system (predisposing patients to cerebral venous clots) and fragile cerebral vessels (possibly via inflammation leading to bleeding).^(27-30)^ These unusual types of strokes have also been associated with COVID-19.^(20,23,26,29- 32)^

While our findings provide important insights into the intersection of COVID-19 and stroke during the pre-vaccine era, they must be interpreted within the context of the study design and the extraordinary circumstances of the pandemic. Our study has some limitations that warrant careful consideration when interpreting the results. First, the absence of a contemporaneous control group of COVID-negative patients with stroke from the same centers and time period limits our ability to definitively attribute the observed high mortality and complication rates solely to COVID-19. The general pandemic-related strains in the healthcare system, including resource reallocation, staffing shortages, and delayed care for non-COVID conditions, may have contributed equally to the poor outcomes observed. However, without such a control group, the direct viral effects cannot be distinguished from the indirect systemic impact of the pandemic on the quality of stroke care. Second, the retrospective study design had inherent limitations, including missing data for certain variables, potential inconsistencies in diagnostic workups across centers, and an inability to establish definitive causality between COVID-19 and stroke outcomes. Third, our study only included hospitalized patients with stroke, inevitably capturing the more severe end of the clinical spectrum of both COVID-19 and cerebrovascular events. Fourth, although the multicenter design enhanced generalizability, it introduced significant heterogeneity in local protocols for COVID-19 treatment, stroke management, and resource availability across participating centers during the chaotic period of the pandemic. Fifth, functional outcome assessments were performed only upon hospital discharge. Consequently, the long-term functional trajectory of these patients, particularly their 90-day mRS scores, remains unclear. This represents an important direction for future research as early discharge assessments may not capture delayed recovery or late complications, potentially underestimating true functional outcomes in survivors. Sixth, we did not collect comparative stroke admission data from the participating centers during the corresponding pre-pandemic period (March- November 2019 or 2020). Such historical control data would have enabled us to assess whether the stroke incidence increased during the pandemic or whether COVID-19 simply represented an additional risk factor. Seventh, we did not systematically collect information on the use of pre-stroke cardiovascular medications (antiplatelets, anticoagulants, and statins), which may influence mortality and functional outcomes. Therefore, future prospective stroke registries should include baseline medication status to better characterize secondary prevention adequacy and the potential protective effects against COVID-19-associated cerebrovascular events. Finally, we acknowledge that the p-value of 0.04 for elevated D-dimer levels independently predicting mortality represents marginal statistical significance; however, we did not correct for multiple comparisons in our exploratory analyses. Consequently, this finding should be interpreted with caution although this association is biologically plausible given the known hypercoagulable state of COVID-19.

## CONCLUSION

In this national Brazilian cohort study, patients with COVID-19 who suffered a stroke predominantly had an ischemic stroke, lower rates of atrial fibrillation, and prior stroke compared to typical non-COVID stroke populations, but significantly higher rates of medical complications and mortality. The D-dimer level was an independent predictor of in-hospital mortality in these patients, reinforcing its role as a prognostic marker for COVID-related coagulopathy. However, contrary to initial expectations, the time from stroke symptom onset to hospital admission was not prolonged during the pandemic. In fact, among our patients, delays were shorter than historical averages, although longer pre-hospital times were associated with more severe neurologic deficits on admission. Notably, these findings must be interpreted cautiously given the substantial limitations of our study design, particularly the absence of a control group and the marginal statistical significance of some predictive factors. Nonetheless, these findings suggest that, while COVID-19 is associated with more severe stroke outcomes, prompt hospital presentation can still be achieved, which is crucial. Our study contributes to the growing understanding of the stroke- COVID-19 interplay and provides a benchmark for outcome expectations in similar middle-income settings. Ongoing vigilance and further research are warranted to mitigate the impact of this pandemic on stroke care.

## Data Availability

Anonymized data, study protocol, statistical analysis plan, and informed consent form will be shared upon request from other investigators after obtaining ethical approval.
