# Supplementary material for: Clinical characteristics and outcomes of SARS-CoV-2-associated stroke: a large multicenter national cohort study
Source: Einstein (Sao Paulo). 2026 Jun 23;24:eAO2027. doi: 10.31744/einstein_journal/2026AO2027 (PMC13399301; doi:10.31744/einstein_journal/2026AO2027)
Supplement: Supplementary Material [file 2317-6385-eins-24-eAO2027-Suppl01.pdf]

SUPPLEMENTARY MATERIAL

Clinical characteristics and outcomes of SARS-CoV-2-associated stroke: a large multicenter national cohort study

João Brainer Clares de Andrade, Vinícius Viana Abreu Montanaro, Mayara Silva Marques, Kristel Larisa Back Merida, Rafaela Almeida Alquéres, Felipe Aydar Sandoval, Millene Rodrigues Camilo, Ahmad Ali El Madjdoub, Letícia Januzi de Almeida Rocha, Vinícius Luiz Cristofolini, Jackeline Viana da Silva, Rafael Paes Alves, Lorena Souza Viana, Felipe Ibiapina dos Reis, Patrícia Beatriz Christino Marinho, Pérola de Oliveira, Pedro Silva Correa de Magalhães, Vivian Dias Baptista Gagliardi, Sheila Cristina Ouriques Martins, Thais Leite Secchi, Octavio Marques Pontes-Neto, Iago Navas Perissinotti, Alan Alves de Lima Cidrão, Deborah Moreira Rangel, Rodrigo Bazan, Gabriel Pinheiro Modolo, Luana Aparecida Miranda Bonome, Felipe Araujo Rocha, Fabrício Oliveira Lima, Adriana Bastos Conforto, Daniela Laranja Gomes Rodrigues, Gisele Sampaio Silva

DOI: 10.31744/einstein_journal/2026AO2027

Table 1S. Univariate analysis of predictors of in-hospital mortality in patients with COVID-19-associated stroke

Variable	Deceased (n=117)	Survivors (n=210)	p value	Included in multivariate model
Demographics				
Age (years), median [IQR]	67 [57- 75]	63 [56- 72]	0.013†	Yes
Male sex, n (%)	65 (55.6)	114 (54.3)	0.916‡	No
Baseline Comorbidities				
Hypertension, n (%)	84 (83.2)	122 (65.2)	0.002‡	Yes
Diabetes mellitus, n (%)	40 (39.6)	69 (36.9)	0.746‡	Yes
Atrial fibrillation, n (%)	8 (7.9)	5 (2.7)	0.080‡	Yes
Previous stroke, n (%)	22 (21.8)	22 (11.8)	0.037‡	Yes
Obesity, n (%)	10 (9.9)	19 (10.2)	1.000‡	No
Current/former smoking, n (%)	11 (10.9)	20 (10.7)	1.000‡	No
Cardiopathy, n (%)	26 (25.7)	29 (15.5)	0.051‡	No
Stroke characteristics				
NIHSS at admission, median [IQR]	18 [10- 24]	8 [4- 14]	<0.001†	Yes
Pre-stroke mRS, median [IQR]	0 [0- 1]	0 [0- 0]	0.001†	No
Ischemic stroke, n (%)	88 (75.2)	180 (85.7)	0.027‡	No
Hemorrhagic stroke, n (%)	12 (10.3)	15 (7.1)	0.441‡	No
Cerebral venous thrombosis, n (%)	7 (6.0)	11 (5.2)	0.976‡	No
Subarachnoid hemorrhage, n (%)	10 (8.5)	4 (1.9)	0.011‡	No
Wake-up stroke, n (%)	8 (6.8)	23 (11.0)	0.307‡	No
Time to admission (min), median [IQR]	226 [95- 610]	270 [120- 730]	0.470†	No
Laboratory parameters (admission)				
D-dimer (mg/L), median [IQR]	3.6 [1.3- 12.4]	1.8 [0.9- 4.2]	0.001†	Yes
Hemoglobin (g/dL), median [IQR]	13.1 [11.5- 14.4]	13.6 [12.1- 14.8]	0.089†	No
White blood cells ($\times 10^3/\mu L$), median [IQR]	10.8 [7.9- 14.2]	9.9 [7.2- 13.1]	0.152†	No
Platelet count ($\times 10^3/\mu L$), median [IQR]	235.5 [171- 301]	265.9 [207- 338]	0.002†	No
Creatinine (mg/dL), median [IQR]	1.2 [0.9- 1.8]	1.0 [0.8- 1.3]	<0.001†	No
Urea (mg/dL), median [IQR]	56.5 [38- 89]	36.5 [28- 52]	<0.001†	No
In-hospital complications				
Pneumonia, n (%)	58 (55.8)	61 (55.5)	1.000‡	No
Acute renal failure, n (%)	54 (51.9)	12 (10.9)	<0.001‡	No
Sepsis, n (%)	49 (47.1)	13 (11.8)	<0.001‡	No
Cardiac arrest, n (%)	57 (54.8)	1 (0.9)	<.001‡	No
Venous thromboembolism, n (%)	7 (6.7)	11 (10.0)	0.539‡	No
Delirium, n (%)	5 (4.8)	9 (8.2)	0.471‡	No

† Mann- Whitney U test (for continuous variables); ‡ χ^2 test or Fisher's exact test (for categorical variables).
IQR: interquartile range; NIHSS: National Institutes of Health Stroke Scale; mRS: modified Rankin Scale.

Variable selection for multivariate modeling:

- Variables with univariate $p<0.10$ were considered for entry into multivariate logistic regression models
- Stepwise backward elimination was used to derive final parsimonious models
- The final models included age, hypertension, diabetes mellitus, atrial fibrillation, previous stroke, National Institutes of NIHSS score at admission, and D-dimer levels.

Model adequacy:

- Mortality model: 117 events / 7 variables = 16.7 events per variable (EPV)
- Favorable outcome model: 95 events / 7 variables = 13.6 EPV
- Both exceed the recommended minimum of 10 EPV for logistic regression
- The variance inflation factors (VIFs) for all variables in the final models were <2.5, indicating no significant multicollinearity.
- Hosmer- Lemeshow goodness-of-fit test: $p>0.05$ for both models (indicating acceptable calibration)

Key findings:

- In total, 15 of 33 tested variables achieved statistical significance at $p<0.10$
- The strongest univariate predictors were in-hospital complications (cardiac arrest, renal failure, and sepsis) and acute severity markers (National Institutes of NIHSS score, D-dimer level, and urea level).
- Classical cardiovascular risk factors showed variable associations; hypertension ($p=0.002$) and previous stroke ($p=0.037$) were significantly associated with in-hospital complications but diabetes ($p=0.746$) was not.
- After multivariate adjustment, only NIHSS and D-dimer retained independent prognostic significance

Important notes:

- Sample sizes vary because of missing data for some variables (especially laboratory parameters)
- Complications listed are those occurring during hospitalization and thus could not be true "predictors" at admission; they are included here for completeness but were not entered into predictive models
- Baseline cardiovascular medication use (antiplatelets, anticoagulants, and statins) was not systematically collected and therefore could not be analyzed.
